# Solidarity and Responsibility in Health Care

**DOI:** 10.1093/phe/phz008

**Published:** 2019-07-04

**Authors:** Ben Davies, Julian Savulescu

**Affiliations:** 1 Uehiro Centre for Practical Ethics; 2 Uehiro Chair in Practical Ethics

## Abstract

Some healthcare systems are said to be grounded in solidarity because healthcare is funded as a form of mutual support. This article argues that health care systems that are grounded in solidarity have the right to penalise some users who are responsible for their poor health. This derives from the fact that solidary systems involve both rights and obligations and, in some cases, those who avoidably incur health burdens violate obligations of solidarity. Penalties warranted include direct patient contribution to costs, and lower priority treatment, but not typically full exclusion from the healthcare system. We also note two important restrictions on this argument. First, failures of solidary obligations can only be assumed under conditions that are conducive to sufficiently autonomous choice, which occur when patients are given ‘Golden Opportunities’ to improve their health. Second, because poor health does not occur in a social vacuum, an insistence on solidarity as part of healthcare is legitimate only if all members of society are held to similar standards of solidarity. We cannot insist upon, and penalise failures of, solidarity only for those who are unwell, and who cannot afford to evade the terms of public health.

## Solidarity in Healthcare

Some healthcare systems, such as the UK’s National Health Service, are described as being grounded in solidarity. Rather than people being responsible only, and entirely, for their own health, the NHS^1^ pools risk through taxation and free-at-the-point-of-use care. Users who are better off (e.g. healthier or wealthier) take on some of the financial risk of worse-off users. This article focuses on the relationship between solidarity and personal responsibility in healthcare. We argue that solidarity can generate obligations, and that failure to meet these obligations can legitimately be penalised. However this can only occur in the right context: both in terms of an appropriate opportunity to choose, and the nature of the society in which such obligations supposedly arise.

**Solidarity and Responsibility**

- Solidarity is a two-way street: a system based on solidarity can require certain kinds of responsible behaviour from participants.
- Failures of solidarity are best enforced when patients have been offered ‘Golden Opportunities’, under conditions conducive to decision-making.
- We should not focus narrowly on solidarity within healthcare. It also matters whether solidarity is practiced in the broader society.

Consider the following cases:

*A 58-year-old man is admitted to hospital following a heart attack, which doctors believe has been caused in part by moderate obesity. The man describes himself as doing ‘as little exercise as possible’, even though he was explicitly warned by his GP five years previously that this inactivity presented a serious risk to his health and turned down an offer of help with getting more exercise.* (Inactivity)

*A 45-year-old woman who smokes twenty cigarettes daily develops chronic obstructive pulmonary disease. Although her doctor warned her about the risks, she was given no support in quitting, and has found it difficult.* (Smoking)

*A 28-year-old man is admitted to A&E following a car accident. He has been driving safely for ten years but later admits that he neglected to put on his seatbelt because he was running late.* (Seatbelt).

Does solidarity recommend treatment, refusal to treat, or something else in cases like these? In part, this will depend on how we conceive of solidarity. At heart, however, the dilemma is this: while each individual is vulnerable, and dependent on society to become well again, they have each made choices that not only impact their own health, but also place costs on society at large. To treat each individual will require resources that could be spent elsewhere and may lead to delayed or cancelled treatment for others who are unavoidably ill. In such cases, the claims of solidarity may seem to pull us in separate directions.

This article argues that a solidary health system can consistently make demands of its members, and impose penalties on them when they are not met. Our view is that solidarity requires that people make only *reasonable* demands of one another. Where people fail to act reasonably, and were well placed to do so, members of a solidary system are entitled to refuse to cover the costs that come from that failure. Finally, we outline some of the constraints on this argument: not all cases where individuals act in ways that affect their health are failures of solidarity.

## What is Solidarity?

In common use, solidarity refers to fellow-feeling and, importantly, mutual support between individuals. This might be because of a shared purpose, as in cases of solidarity amongst striking workers. But it may involve taking on a goal because of one identifies with those already involved. While solidarity has been adapted in different ways by various traditions (Prainsack and Buyx, 2011; Prainsack and Buyx, 2017: 19–42), one if its most prominent contemporary uses is to invoke ‘emotionally and normatively motivated readiness for mutual support’ (Laitinen and Pessi, 2014: 1). This includes a willingness to promote others’ interests, or the interests of the group, even at personal cost.

Solidarity can act as a descriptive concept, explaining the emergence of norms or institutions. Some argue that the NHS was founded in a spirit of solidarity following the Second World War.^2^ It can also act as a normative motivation, where group membership generates what Shelby (2002: 68)^3^ calls ‘robust solidarity’: rather than merely describing practices as solidary, robust solidarity requires that group members feel obligated to act in certain ways as a result of solidary bonds. Since the NHS is funded by taxation and free at the point of use (for many),^4^ it involves mutual support between members of UK society. On this basis, one might think that each of the individuals involved in Inactivity, Smoking and Seatbelt have a claim to treatment based in solidarity. They are vulnerable members of the relevant society and their fate is at the discretion of a system that was established to help people in just their positions.

Solidarity’s association with individual action (Buyx and Prainsack, 2017: 43–48) may make it seem an inappropriate label for a complex institution like the NHS. But while solidarity may be most obviously expressed in direct contact between individuals, it can also be expressed by active support for policies that involve the sharing of risk and benefit; this includes concrete political action such as voting for solidarity-supporting political parties; campaigning for policies that support others’ interests as well as one’s own; and participating in (well-directed) socio-political schemes that support solidarity-enhancing institutions, e.g. paying one’s ‘fair share’ in taxation.

While solidarity cannot come from institutional design alone, we may nonetheless describe as solidary *to some degree* principles and institutions that both aim to enforce solidary norms, and which are supported for solidary reasons by at least some participants. This would, in Nagy’s (2002: 329) terms, be an instance of ‘thin’ solidarity, in contrast with the ‘thick’ solidarity that is generated in a bottom-up way by ‘substantive’ moral agreement. At the macro level, then, institutions and practices can be more or less solidary depending on the proportion of participants who support them for solidary reasons and, depending on whether they are intended to be derived from, and supportive of, solidary relationships among participants.^5^

In addition, it is sometimes legitimate to enforce social arrangements with the aim of fulfilling solidary obligations, even if solidary feeling and action is low within a community. Whether the resulting arrangement genuinely fulfils obligations of solidarity depends in part on how we conceive the relationship between the descriptive and normative facets of the concept. Our view is that we can criticize certain institutional arrangements for a failure to show solidarity because they govern social relationships that *should* be at least somewhat solidary in nature.^6^ Robust solidarity governs obligations that group members feel as a result of their membership, and we assume that this feeling can be more or less accurate depending on the circumstances: sometimes people will fail to feel solidary obligations, even though they do in fact exist. Recent writing on solidarity (West-Oram and Buyx, 2017: 217–218; West-Oram, 2018) emphasises the potential for *building* solidarity (including at the global level) out of self-interest. While state institutions cannot create solidarity inorganically, they can ‘provide the social bases for realizing relations of … solidarity’ (Krishnamurthy, 2013: 134).

A critical element of solidarity is its characterisation as ‘we-thinking’. This distinguishes it importantly from charity, which is purely other-directed. In a solidarity-based arrangement people not only give to others, but are entitled to expect something back. Again, this is often derived from shared group membership, or at least some shared characteristics or interests. As Prainsack and Buyx argue (2017: 53–54), solidarity requires the recognition of similarity, and its prioritisation (at least in one respect) over difference (see also West-Oram and Buyx, 2017: 216, who emphasise solidarity’s focus on the similarities between individuals, compared with charity’s focus on, e.g. wealth differences).

In our view, this means that a principle of solidarity does not direct us uniformly towards treatment in our test cases. Although individuals operating in a solidary system have *claims on* others, and the system in which they operate, since solidarity is reciprocal people also have *obligations to* others and may have obligations to behave in certain ways within that system. This suggests two relevant questions. Have the individuals in our cases failed an obligation of solidarity? And if they have, is the obligation of the kind where failure warrants a penalty within that system? This is the topic of the next section.

## Obligations of Solidarity

Solidarity’s characterisation as ‘we-thinking’ seems to us to require a degree of reciprocity, and hence obligation. One might object to this claim, noting that some solidary actions do not appear to involve reciprocity. For instance, Prainsack and Buyx imagine a low-level example of solidarity, lending a fellow passenger one’s phone when you are both stranded at the airport. Similarly, we can surely experience solidarity with those who are *unable* to reciprocate, at least in kind. How, then, can we suggest that solidarity *requires* reciprocity?

It is thus important to clarify the idea of reciprocity in three ways. Firstly, reciprocity does not require an identical give and take. Rather, it requires ‘playing one’s part’. Particularly at the institutional level, where thousands or millions are involved in a solidary institution, solidarity cannot require that everyone gets back exactly what they put in; indeed, as we argue below, such a transactional institution is not genuinely solidary at all.

Secondly, as the phone example makes clear, solidary reciprocity need only be hypothetical. In lending your phone to a fellow passenger there is an implicit assumption, we suggest, that they would do something similar for you or someone else in a similar position. If you had evidence to the contrary (e.g. you had just seen them refuse to lend some change to a fellow passenger to buy a drink), you might feel less inclined towards solidarity with them.

Finally, our claim that solidarity is reciprocal does not mean that we disagree with Prainsack and Buyx’s claim (2017: 62) that solidary action cannot be *solely motivated* by the expectation of reciprocity. Rather, we claim only that solidarity generates obligations to contribute to solidary systems and institutions, dependent on one’s ability.

As well as reciprocity, solidary also requires a commitment to action. Taken together, these commitments make it clear why solidarity-based institutions may demand a degree of personal responsibility from their participants. On the other hand, a willingness to abandon those who make poor choices with respect to their own health seems to be the antithesis of solidarity. We can agree that those who knowingly place avoidable burdens on a public health system have in some cases failed an obligation of solidarity, without concluding that they have thereby forfeited their solidarity-based claims. We therefore need to consider what the relevant obligations are, and whether they warrant penalties in cases where they are violated.

One candidate for a relevant solidary obligation is the obligation not to externalise the costs of one’s decisions in ways that burden others. In its simplest form, though, this cannot be correct. For the very idea of solidarity is precisely that we share, to some extent, in one another’s burdens. Buyx and Prainsack (2011) argue against using solidarity to ground health-related liabilities on such a basis. They suggest that any attempt to do so will focus on easily identifiable^7^ failures of responsibility, obscuring the fact that all of us make choices that raise the risk of some health burden or other. It would be unfair to refuse to externalise some kinds of freely chosen health burdens, and not others. Indeed, such a selective policy risks narrowing the range of conceptions of the good life.^8^ We ought in principle to support choices which, though entailing risks, either plausibly aim at well-being or which are fully autonomous on either a Millian or Kantian conception.^9^

In our view, an argument for holding people responsible based on solidarity should identify types of burdens that people cannot expect others to shoulder in the name of solidarity. One option is to adopt a luck-based focus.^10^ Translated to the language of solidarity, this suggests that solidarity requires others to accept our externalised costs when those costs arise as a result of bad ‘brute luck’, i.e. misfortune due to unforeseeable accident, or misfortune imposed on us by others, but that it does not require that society externalise the costs of burdens that are due to bad ‘option luck’, i.e. outcomes that were foreseeably avoidable by the agent.^11^ But this seems to misclassify many cases. A wide range of options are avoidable, will involve some health burdens, and yet are entirely reasonable to choose. This includes choosing to meet existing moral obligations, choices where alternative options also have significant costs, and choices which, despite being risky, also offer considerable prospective benefits. It would be unreasonable, and in violation of solidarity, to refuse to support people who make such choices.

Instead, we suggest that solidarity licenses sanctioning people who externalise costs to others when this externalisation is *unreasonable*. Solidarity requires us to take up common cause with those who are suffering only if they show a reciprocal concern for us, so long as they are able to do so. Those who choose to impose unreasonable burdens on others—or choose, unreasonably failing to consider the burdens on others—have failed to show this reciprocal concern.

In practice, it will be difficult to determine whether a choice is aiming at a reasonable conception of the good life^12^ or is fully autonomous. However, we may restrict our scope to those who are responsible for their own health burdens under certain choice conditions. Even if we all make decisions that ultimately impact our health, it is not true that we all make such decisions under the relevant set of conditions, i.e. conditions that are sufficiently conducive to well-considered, uncoerced choice, and which are unreasonable given the context.

Prainsack and Buyx’s concern holds only if all health-impacting choices demonstrate equivalent failures of solidarity. This is not obvious. Some behaviours, despite carrying health risks, also carry considerable health benefits. If I end up worse off after following medical advice, this does not constitute a failure of solidarity in the same way as a decision to ignore, or to fail to attend to, medical advice. Similarly, personally unhealthy choices that appear to violate obligations of solidarity may be the only way to fulfil other obligations: for instance, working long hours at great cost to one’s health to be able to feed one’s children. We thus cannot move from the fact that we all make choices that harm our health to the claim that it is unjustified to pick any subset of those choices as appropriately subject to substantive responsibility. Some such choices are more reasonable than others. What this does suggest, however, is that we cannot consider solidarity in health-based decisions in isolation from our broader social context, or the conditions of choice.

It is worth saying something at this stage about the relationship of solidarity to justice. Since we are concerned with the imposition of unreasonable costs, it may seem that the real topic of our discussion is distributive justice. There are three things to say about our view on this. Firstly, the requirements of justice are affected by solidarity. Although solidarity is not itself always obligatory, the existence of solidary relationships affects the types of entitlements people may claim on grounds of justice. Secondly, we earlier suggested that obligations of solidarity may exist even in the absence of relevant feeling. If people stand in certain relations to one another (e.g. the relationship of fellow citizen), justice may itself demand a level of solidarity. While our interest is in exploring the parameters of what solidarity requires of us, this is indeed intimately related to justice. Yet the centrality of justice to our discussion does not negate the importance of solidarity.

Finally, however, we accept that justice is in some sense prior to solidarity. This is both because minimal standards of justice are a prerequisite for solidarity (e.g. Krishnamurthy, 2013), and because justice sets boundaries on what solidarity can demand of us. However, we assume that in the allocation of health care resources, we cannot treat everyone who would benefit, and that justice may not offer comprehensive, decisive instruction on which individuals should lose out. This means that considerations of justice (e.g. claims of reparation on the basis of past injustice, or claims that one is unconditionally entitled to a minimal amount of care) may constrict which conditions are properly subject to penalties; but where at least some patients must lose out, and justice thus cannot preclude any particular individual from facing additional burdens, the considerations of solidarity that we outline are relevant.

## The Conditions of Penalising Solidarity Failures

Some choices that affect our health meet the highest standards of autonomy: they are made with full knowledge of consequences, using well-functioning rational capacities, in circumstances where a reasonable array of options is available. The case of Inactivity^13^ meets these criteria. By stipulation, the patient could exercise more but chooses not to, and does so with reasonable understanding of the potential risks over a considerable length of time.

Other choices fail to meet these standards. Many behaviours that are cited as leading to ‘lifestyle-related diseases’, including our case of Smoking, are chosen without explicit coercion, but under strong social influences beyond individuals’ control, and they are often only threats to health when part of unreflective, long-term habits, driven in part by advertising and other lifestyle constraints and pressures. Other health-affecting choices are impulsive errors of judgement or mistakes. Seatbelt is a case of this type.

Smoking and Seatbelt present problems for many standard analyses of responsibility because they exhibit a mixture of failure and success with respect to features that make decisions responsible. For instance, neither is chosen after a period of reasonable reflection,^14^ nor are they (we stipulate) endorsed by second-order desires.^15^ Smoking may be in character^16^ for our patient if that is understood as concerning what a patient *typically* does, but not if we understand character in terms of higher order desires. Seatbelt is out of character in both senses. While there are cases of inactivity that also involve these barriers (see fn7), the patient in our case, we stipulate, faces more favourable conditions. He faced many opportunities, and no special barriers, to doing more exercise, including having the spare time and money such that doing so would not be burdensome. He also, we imagine, reflected on whether to exercise, knowing its effect on his health. But he decided that he would rather avoid exercise and risk poor health.

One argument in favour of solidarity-based penalties is that participation in solidary practices or institutions generates obligations, and failure to meet those obligations can justify either exclusion from the practice, or penalties within it. A system of tax-funded healthcare is such a practice. For instance, Buyx (2008) suggests that, while solidarity places a constraint on the degree to which we may hold people substantively responsible for their own health, it does not ground an absolute objection to the inclusion of personal responsibility in healthcare, since solidarity cuts both ways. If my decisions demonstrate a failure to show due regard to other members of my community, I fail to demonstrate appropriate solidarity.

In none of our cases is there an intention to betray solidarity or violate obligations. People don’t smoke or drive unsafely with the health budget in mind. If anything, this absence is even starker in cases involving neglect. The smoker might consider the alternative and intentionally reject it. Not so the person who neglects to put on their seatbelt because they are distracted and in a hurry: even their failure to act appropriately seems unintentional. With respect to solidarity, then, these cases are all marked not by intentional refusal to fulfil an obligation, but by failure to consider that there is such an obligation at all, and possibly by further failures of intention as well.

However, that an outcome is due to someone’s failure does not preclude responsibility for that outcome; nor does failure to consider an obligation exempt us from it. Raz (2010) argues that people are responsible for actions that are ‘governed’ by our rational agency, even when that agency fails (e.g. because we failed to pay attention). Indeed, many failures of practical rationality (doing what you should do) are attributable to failures of theoretical rationality (believing what you should believe).^17^ And many failures of theoretical rationality are blameworthy since the agent is responsible because she should have known (or believed) better. There are cases where it is not only legitimate but required to apply substantive responsibility to failures of intention. If a company fails in implementing appropriate safety measures, leading to an accident, it cannot escape liability by protesting that it did not *plan* the accident. If I cause a car crash because I am distracted, I cannot escape criminal penalties or compensation for victims for this reason alone. In both cases, an obligation exists, and failure to fulfil it is not intentional, but negligent.^18^

One problem with many potential ways of involving responsibility in healthcare is their excessive simplicity. This applies to the behaviour required to trigger a penalty: one bad habit, or even one mistake, is sometimes seen as enough to justify considerably different treatment. This problem also applies to the finality of the decision to penalise. As Eyal (2013) notes, responsibility penalties often set patients up to fail by conditioning ‘the very aid that patients need to become healthier on success in becoming healthier’.

This latter issue has led to several related proposals around how we should think about responsibility in healthcare. Feiring (2008), for instance, distinguishes between ‘backward-looking’ and ‘forward-looking’ responsibility, suggesting that while we cannot penalise patients for their past irresponsibility, we can set conditions for future healthcare. However, as Albertsen (2015) notes, there is something paradoxical about this proposal: even if the conditions that we set upon commencement of treatment are forward-looking at that point, they *become* backward-looking if we later penalise patients for failing to meet them.

More promising is the idea that responsibility can be invoked only when patients have refused a ‘Golden Opportunity’ (Savulescu, 2018) and appropriate choice conditions have been determined and set. Golden Opportunities involve patients being given concrete, health-promoting behavioural changes. Importantly, this includes the stipulation that patients must be given ‘considerable support’ in their lifestyle change: merely being told that a behaviour is unhealthy, as in Smoking, is not enough.^19^

What is most relevant about Golden Opportunities is not whether the relevant behaviour is in the past or future, but whether it is performed under circumstances that are conducive to responsible choice. Additionally, Golden Opportunities must be ‘realistically adoptable’. We cannot demand that the long-distance haulier takes 10,000 steps every day, or that the single parent working two jobs cooks fresh food every evening. Part of the spirit of solidarity involves recognising that not everyone can contribute to keeping collective costs down in the same way, or to the same degree. In this sense, while Prainsack, Buyx, and West-Oram are right to say that solidarity is centred on similarity, it is not possible to practice genuine solidarity without recognising difference.

However, it is also important to acknowledge that patterns in the barriers people face in making healthier choices. Genetic predisposition may mean that an individual faces weight gain when following what, for others, would be a healthy lifestyle. In addition, there is considerable evidence that poverty and social inequality contribute to poor health (e.g. Marmot, 2005). This may occur in various ways; most pertinent for our purposes is the recognition that poverty and inequality bring with them reduced opportunities. People who face poverty have less time, less money and less energy to make healthy choices. A judgement that an opportunity is realistically adoptable, then, must take account of the structural barriers patients may face in changing behaviour.

One of us (JS) has previously suggested that a genuine Golden Opportunity is one where there is no overall trade-off in value either because there is a reduced risk for the same value—for instance, swapping cigarette smoking for vaping retains the pleasure of smoking—or the same risk for increased value. However, since our justification for introducing responsibility as a limited rationing tool is solidarity, it is acceptable to allow some value loss overall, since solidarity involves a willingness to accept some personal costs for the benefit of others (Prainsack and Buyx, 2017: 52–53). If a patient finds vaping less pleasurable than smoking, but the health risk is significantly lower, it is reasonable to require this as a behavioural change. Once we set these parameters, Buyx and Prainsack’s concern that *everyone* makes decisions that externalise costs in a way that is relevant to solidarity looks far less likely to be true. For when confronted with a clear, health-improving option, and offered support in making it, many will choose to take that option.^20^

Returning to our three cases, our view is that only Inactivity meets the requirements set by Golden Opportunities. Golden Opportunities do not apply in cases of impetuous mistakes in reasoning, as in Seatbelt, even if these are autonomous and avoidable. While one might face a Golden Opportunity with respect to smoking, the patient in Smoking has not been offered support to help her quit, instead struggling with it herself. Depending on the nature of the help offered, the patient in Inactivity may be considered to have been offered a Golden Opportunity. The only element missing is his doctor making it clear that the offer of help is subject to a penalty if refused.

Since Inactivity involves a patient who is obese, it is important to reiterate that it is not obesity itself that entails a Golden Opportunity. We accept the considerable role of environment and genetics in obesity. This is one reason that Golden Opportunities are structured as they are. Results are important for Golden Opportunities: a patient only faces a Golden Opportunity when there is good reason to believe that the relevant change will improve their health. But patients are not thereby judged *by* the health results, but by the behavioural changes they adopt. If the patient in Inactivity makes a sincere effort to exercise more, that is sufficient for his having taken his Golden Opportunity. Similar points apply to our claim that the patient in Smoking could have faced a Golden Opportunity if offered support with quitting. What patients are held responsible for is not solely the health-affecting behaviour, but the decision whether to accept effective help overcoming it. Finally, we should stress that including personal responsibility within a healthcare system does not preclude acknowledging the significant role of other factors on patient health.

A further condition concerns the type of penalty that is appropriate. Eyal’s concern about removing the means patients need to reach the goal they are penalised for not achieving applies most obviously to patients who have not had opportunities to adopt healthier behaviours. However, it may also speak against the relevant penalty being straightforward denial of care. One alternative is to recover costs pre-emptively through taxation or mandatory insurance (e.g. Cappelen and Norheim, 2004; Bærøe and Cappelen, 2015).

There are two obvious worries about this. Firstly, not all unhealthy behaviours are taxable: some are illegal; some behaviours that can be subject to mandatory insurance if done in licensed ways (such as extreme sports) can be practiced outside approved contexts; and some would require excessive monitoring to properly track.^21^ In this context, it seems clear that a tax/insurance approach can only work as a best-case scenario, not as a catch-all. In cases where costs cannot be recovered pre-emptively, other approaches may be justified. Nonetheless, we still need not turn immediately to outright denial of care. Other possible approaches include partial covering of health costs (subject to ability to pay), and lower priority on waiting lists.

The second worry is that the overall burdens (including costs involved in public education and dissuasion, as well as in medical research funding that could be directed elsewhere) of some risky activities are so great that no realistic tax or insurance could cover the costs. If we cannot recover all relevant costs pre-emptively, and no other penalties are applied, then responsible individuals will still free-ride to some extent on a solidary social scheme.

However, this concern only holds if obligations of solidarity apply to every one of our actions, and if they are unrestricted in terms of the costs that can be imposed for those who violate their obligations. If there are limits on both these factors, an inability to recover the total costs which result from a particular behaviour does not invalidate pre-emptive cost recovery as a reasonable option. For instance, assume that realistic alcohol taxes cover only 75 per cent of the costs associated with excessive drinking, because higher taxes would drive people to the black market, lowering overall tax revenue while leaving alcohol-related health problems unchanged. If the obligations arising from solidarity needed to cover 100 per cent of costs from solidarity-violating behaviour, this would leave 25 per cent of costs unjustifiably remaining. But if solidary obligations require only that people who make unhealthy choices (under the right conditions) cover *some* of the associated costs, this incomplete recovery may not be a problem.

In fact, solidarity does not demand that those who fail their solidary obligations must cover all relevant costs. This would apply only if a single violation of solidary obligations justified excluding someone from solidarity-based institutions entirely. Segall (2005: 339) contrasts genuinely reciprocal arrangements (of which we assume solidarity is one type), with schemes of cooperation that do not generate public goods, and so from which individuals can be excluded. As Segall argues, public goods are non-excludable, and so ‘an obligation to contribute applies *because* the fruits of social cooperation have “a quality of normative non-excludability”’.

To put this in the language of solidarity, it only makes sense to talk of participants failing their solidary obligations if we are operating a system that is to some degree independent of individual participants’ decisions to opt in. If our cooperative scheme were structured so that what you put in is what you eventually get out, there could be no obligation to other participants to reduce costs. This is because if you act in a way that means you put less in, the only outcome is that *you personally* get less out, and because the absence of public goods means that each individual can decide whether to participate or not.

A system based on solidarity does not work like this. Solidary obligations arise because of relationships and similarities that already exist between individuals. It is *because* we cannot abandon people that they in turn derive obligations to play their part by not overly burdening the system we share: it would be unreasonable of them to burden that system, since it is a system the rest of us cannot ethically—and perhaps even practically—opt out of. As such, the failure of pre-emptive taxes and insurance to cover all costs associated with a behaviour does not undermine this proposal, because a solidary system does not require precise matching between payments into a scheme and the benefits one gets out.^22^ Solidary obligations also do not require perfect compliance in all behaviours. Individuals can behave self-interestedly, and perhaps sometimes selfishly or negligently, on some occasions and still conform broadly to their obligations of solidarity. Individuals who are part of a ‘we’ are also still individuals; even in a solidarity-based system, a balance must be struck between the demands of solidarity, and the rights of individuals not to have to behave perfectly.

There are two general arguments against solidarity requiring absolute compliance. The first, assuming that group membership is the basis of solidary obligations, is that people have multiple such affiliations that may compete with one another. No single system or group can demand perfect compliance. The second argument connects to some extent with Buyx and Prainsack’s sceptical view of solidary penalties. Even with a limit on the kinds of choices for which we can be held responsible, most people will not comply perfectly with responsible behaviour even if they are motivated by solidarity. We are all subject to temptation, to weakness of will, and to moral fatigue.

This does not mean that we can define, in the abstract, a precise level of solidarity that constitutes meeting one’s solidary obligations. If solidary obligations arise from the bonds and connections involved in particular institutions and social practices, exactly what is required will depend on the nature of the group or scheme, and perhaps even the views of those involved. Nonetheless, requiring some minimal degree of solidarity through the kind of healthcare planning found in Golden Opportunities does not require perfection from patients, but only a minimal or sufficient commitment to taking on some of the burdens of promoting their own health.

A solidarity-based system that relies on Golden Opportunities places conditions on full inclusion in the healthcare system. One potential criticism is that these policies penalise only *some* individuals for failure to comply with certain behavioural requirements. Nobody is responsible for their poor health in a vacuum; social conditions and biological predispositions both play significant roles. For instance, if we take two moderate smokers, only one may be unlucky enough to be genetically predisposed to developing cancer from their level of smoking. Since Golden Opportunities kick in only once someone risks developing a significant health problem, only the unlucky will be placed at risk of exclusion.

A related problem stems from wealth. Few states have placed outright bans on private healthcare and so most public healthcare systems allow wealthier patients the option of opting out of the public system. One might worry that Golden Opportunities therefore risk introducing a system whereby poorer patients have a duty of solidarity enforced through potential denial of medical care, but wealthier patients can engage in risky behaviours and then refuse Golden Opportunities at no personal cost. This will also raise worries about the targeting of certain kinds of already stigmatised behaviours and groups (e.g. Friesen op cit), and of targeted moralisation, where an already less well-off, or vulnerable, minority are morally blamed for failing to maintain adequate health (e.g. Brown, 2018).

These issues highlight the importance of the broader social environment in thinking about solidarity. No healthcare system exists in isolation. To avoid inequitable distribution of burdens, any attempt to enforce certain healthy standards of behaviour for those who are already in poor health (or at risk of it) must operate within a broader social system that also exhibits solidarity.

However, one might worry that any policy which warrants exclusion of some individuals from healthcare presents practical risks. Although we endorse a careful consideration of individual circumstances, in reality two types of worry arise. First, any new criterion by which people can be excluded from healthcare, or levied additional charges, is a criterion that can be misused either through error, or intentionally by governments or insurers keen to save costs. Second, a more general worry is that focusing on failures of reciprocity might undermine solidarity in general, because of its emphasis on difference, rather than on the similarity that grounds solidarity. Although we have endorsed strict limits on the conditions under which responsibility can lead to penalties, one might worry that it is bound to increase stigmatisation of already vulnerable groups.

We accept these concerns as significant and legitimate. With respect to miscategorisation, it is a requirement of any fair system that aims to hold individuals responsible that it is open to challenge, and that resources are provided for such challenges. In addition, the danger of misuse of responsibility may suggest limits on the type of penalties that are appropriate. In particular, our view is that total denial of care is unlikely to be a suitable penalty for this reason. Further, if a solidary system does aim to make use of the concept of responsibility, we must also recognise the importance of *increasing* the opportunities people have to make healthy decisions. The idea of support inherent in Golden Opportunities does not only relate to advice, but to funded resources and programmes of which citizens can make use to improve their health. Our claim, however, is that if people have the opportunity to make healthy choices, it is sometimes reasonable to hold them responsible for choosing not to.

Perhaps the more fundamental challenge comes from the idea that invoking responsibility, whether appropriate or not, risks undermining responsibility through its focus on difference and division. Again, we acknowledge the risk. However, it is worth noting a countervailing risk. If there is a general perception that many individuals are behaving unreasonably, this may itself weaken the ties of solidarity. Moreover, as we outline below, an acknowledgement of difference is consistent with a simultaneous emphasis of similarity.

One way to emphasise similarity, which also responds to some extent to the concern about genetic and social luck raised above, involves expanding the idea of Golden Opportunities by recognising that they may apply to many more of us than just those who indulge in the standard ‘bad habits’ or who have serious health problems. Recall Buyx and Prainsack’s concern about using solidarity as a rationing tool: we all make choices that impact our health. Most of us, then, will have the opportunity to make changes to our lifestyles that will give moderate health benefits, or at least maintain our current level of health.

A focus on solidarity can help us to see that what is relevant is not simply how much of a financial cost an individual’s health needs place on the health service, but on whether individuals are willing to make moderate, reasonable sacrifices to avoid burdening others. The incorporation into a national health service of a Personal Health Plan, developed in coordination with the relevant patient, in recognition of their own limits and aspirations, is one potential way to expand the range of the basic idea of the Golden Opportunity. Such plans must be developed with recognition that health is not the sole value for anyone, nor the primary value for many. Our demands on one another cannot be that we optimise our health. For instance, some individuals may reasonably prefer to risk physical injury in pursuit of sporting achievement. Others may risk other kinds of health problems by adopting a sedentary lifestyle because they value work that requires long hours at a desk. Both individuals may be able to lower the relevant risks they face, and to improve their behaviour in areas that are less fundamental to their central goal, such as diet. As such, while Savulescu’s original examples of Golden Opportunities are rare, our view in this article is that the structure of the Golden Opportunity can in fact be made available to a significant proportion of individuals.^23^

Finally, we turn to the potential for the better-off to escape any potential penalties in healthcare.^24^ This means that demands of solidarity must extend beyond the healthcare system. It is hypocritical to insist that some sections of the population engage in ‘we-thinking’ (and its associated behaviour) without also insisting on such thinking for the rest of the population. Any solidarity-based enforcement of personal responsibility in healthcare, then, should come in tandem with policies designed to penalise failures of solidarity among the wealthy, e.g. through tax avoidance, exploitative working conditions, pollution, and so on. To punitively pursue solidarity only in a way that will disproportionately affect the more vulnerable is to pursue solidarity in name early, and its opposite in practice. Moreover, if private patients are not fully internalising the costs of their choices, then the same obligations of solidarity would apply.

Concerns about equitability also add a further reason to avoid absolute denial of basic care as a penalty. Even if one thinks that denial of basic care can be a legitimate penalty for failures of solidary obligations, it cannot be legitimate to have a system that in practice denies basic care only to those who cannot afford private care. It may also point us towards certain ways of implementing other forms of substantive responsibility. For instance, if substantive responsibility requires that one contributes financially to the costs of one’s care in a way that those who are not responsible for their medical needs are not required to, such contributions may need to be weighted according to ability to pay, or kick in only when doing so would not affect a person’s basic needs.

One might think, on the other hand, that those who *can* afford private healthcare might face harsher penalties than those who cannot, including being excluded from the solidarity-based system altogether. Whether this is an appropriate solution will depend on whether private healthcare is consistent with a solidarity-based approach. For instance, some argue that pushing the wealthy out of benefits systems leads to an overall decrease in public support for those systems. To paraphrase social researcher Richard Titmuss (1967) (cited in Alcock *et al.*, 2001), the worry is that public services for the poor end up being poor public services. However, this will depend on the level at which exclusion begins. It is one thing for a service to be limited to the very worst off, and hence fail to benefit the majority; it is another thing for it to be limited to all but the very best off (and then, only those in that group who behave irresponsibly), and thus accessible to most.

## Conclusion

Our three cases—Inactivity, Smoking and Seatbelt—all involve people making choices that impose costs on the public purse which are, in some sense, avoidable. In some cases, such choices may violate obligations that arise from being involved in a solidary arrangement and do so in such a way as to warrant penalties. However, we have argued that a violation of solidary obligations requires more than avoidability: it requires both that the risk taken is unreasonable, and that it was made under conditions that are conducive to autonomous choice, as embodied in being offered a Golden Opportunity. Inactivity meets these conditions, so long as his refusal of help is made in knowledge of the potential penalties. Finally, we argued that imposing solidary obligations through penalties requires looking to the broader social environment. Only if solidarity is practiced and enforced here, particularly with respect to the most secure, is it reasonable to enforce it amongst those who need medical care.

## Footnotes

1. The following discussion is based primarily on a system like the NHS, which is what we might call ‘fully solidary’. Clearly this is not the only possible way to structure a health system. One might, for instance, have a system which, although funded largely by progressive taxation, expects minor co-payments from patients at the point of service (e.g. £1 every time one visits a GP). Such payments could be capped for those with chronic conditions, and waived for the very worst-off. Such a system might encourage solidarity by getting patients to see themselves as contributing directly to the health system. But there is, of course, a danger of putting off patients who need medical attention, but are concerned about the cost. The fact that a health system requires some up front payment from patients does not prevent it from being solidary. So long as there is some subsidisation of the worse-off by the better-off, we have a somewhat solidary system. Our view applies, at a minimum, to the sections of a healthcare system that are governed by such solidary practices.

2. Tallis (2016); Broxton (2017); Heath (2018). Though see Molloy (2018).

3. See also Taylor (2015)

4. NHS services are subject to an Immigration Health Surcharge for many people applying to enter or remain in the UK. See: www.gov.uk/healthcare-immigration-application.

5. For instance, Fenger and van Paridon (2012: 51–2) distinguish between ‘individual’ and ‘institutional’ solidarity (in Reinventing Social Solidarity Across Europe), the latter of which ‘involves a certain amount of pressure, a certain degree of organisation and the presence of a set of formal or informal rules’. See also Prainsack and Buyx’s discussion of different ‘tiers’ of solidarity, the most general sometimes involving state coercion to implement solidary practices (2017: 54–7). Even if such institutional practices are not backed up by explicit feelings of solidarity from the majority of the population, they typically rely on the ‘willingness of individual persons to carry costs to benefit others’ (West-Oram, 2018: 581). Prainsack and Buyx (2017: 36) note the distinction between solidarity as an essentially voluntary ‘community value’, and as ‘system value’, enforceable by law.

6. For instance, Fraser (2008: 150–3) argues that solidarity can exist both as a result of a subjective *sense* of solidarity, but also due to causal interdependence. In the latter case, we might say that given the existence of such dependencies, individuals and states have obligations of solidarity even in the absence of any relevant sentiment.

7. See also Friesen (2016).

8. Hope *et al.* (2008)

9. Savulescu (2007)

10. E.g. Arneson (2000); Cohen (2011); Lippert-Rasmussen (2015); Segall (2016)

11. See Vallentyne (2002) for a discussion of various ways of understanding the distinction.

12. Some think, for instance, that elderly patients behave unreasonably if they expect life-extending treatment past a particular age (e.g. Callahan, 1995). See Overall (2005) for response.

13. This judgement is based not on the type of behaviour—there are many cases of inactivity that are not easily avoidable—but on the circumstances of choice.

14. Vallentyne (2008)

15. Frankfurt (1971); Dworkin (1988)

16. Christman (1991).

17. Savulescu and Momeyer (1997).

18. One thing that does mark these cases out, however, is that they fall within pre-existing institutional or social frameworks where it is reasonable to *expect* to be penalised for such failures. Any case for substantive responsibility being attached to failures of solidary obligations must occur within such clear institutional framework. In other words, it should be clear to individuals who might face penalty that this is a possibility.

19. One might worry here that Golden Opportunities seem likely to occur quite rarely, and so may be of limited use in a national health care system. We suggest a potential expansion of the concept below.

20. It is worth noting in this context that the question of costs is more complex than it may seem. For instance, West-Oram (2018: 582) notes that a solidary approach to healthcare can sometimes offer benefits even to net financial contributors, such as the effect of herd immunity through free vaccinations. Assume that offering relevant vaccinations (backed up by sufficient information on their importance and safety) to any patient who visits a doctor or hospital is a Golden Opportunity. Some adult patients who refuse this opportunity may then become unwell. Even if it is permissible to penalise these patients, it may be overall better to treat them for free if their condition is both infectious and serious.

21. Bærøe and Cappelen, op cit 838; Wolff (1998).

22. This is also the reason that the patients in our cases cannot, for instance, appeal to the fact that they have contributed towards the funding of the NHS, thereby supporting others in *their* unhealthy choices. Such a view would imply that those who have not paid in have less right to behave unreasonably than those who have—or, that those who have not paid in as much have less right than those who have paid in more.

23. It is also important to emphasise that holding people responsible for certain decisions need not involve a judgement of their character. Rather, the claim is that solidarity entitles us to expect people to impose only reasonable costs on others.

24. An alternative is to prevent individuals from buying direct health services outside of the basic package provided by the state (e.g. Gutmann, 1981: 553; Walzer, 1983: 90).
